# Comprehensive Evaluation of the Safety and Efficacy of MOSY PHYCOLLREPAIR: A Novel Fermented Black Bee Honey‐Derived Cosmetic Ingredient

**DOI:** 10.1111/jocd.70395

**Published:** 2025-08-21

**Authors:** Bingying Sui, Junyuan Chen, Xueping Chen

**Affiliations:** ^1^ Hangzhou Naimo Cultural Media co., Ltd. Hangzhou China; ^2^ Vitargent (International) Biotechnology Limited Hong Kong SAR China; ^3^ Centre for Biotech big Data Research & Development Research Institute of Tsinghua Huangpu District Guangzhou China

**Keywords:** anti‐aging, collagen regeneration, MOSY PHYCOLLREPAIR, skin barrier, zebrafish embryo

## Abstract

**Background:**

MOSY PHYCOLLREPAIR is an innovative cosmetic ingredient extracted from fermented blank bee honey, specifically engineered for dermatological applications.

**Objective:**

This study seeks to thoroughly evaluate the safety and diverse efficacy profiles of MOSY PHYCOLLREPAIR within cosmetic formulations.

**Methods:**

The safety of MOSY PHYCOLLREPAIR was determined through zebrafish embryo developmental toxicity assays. Its dermatological benefits were assessed via a comprehensive series of in vivo zebrafish embryo tests, encompassing antioxidation, anti‐inflammation, modulation of type I collagen, elastin, and sirt1 gene expression, wound healing, skin barrier integrity, UV protection, and skin whitening.

**Results:**

MOSY PHYCOLLREPAIR demonstrated non‐toxicity to zebrafish embryos at concentrations up to 3.08 mg/mL. It showed significant efficacy across multiple parameters: 29.10%–62.48% anti‐ROS activity, 11.08%–27.84% anti‐inflammatory effect, 60.25%–295.67% ATP‐enhancing efficacy, and 6.52%–17.80% melanin inhibition at concentrations ranging from 25 to 500 μg/mL. It also upregulated the expression of type I collagen genes *col1a1* by 87.67% to 281.61%, *col1a2* by 54.08% to 165.14%, astin gene *elna* by 55.81% to 174.03%, and *sirt1* gene by 60.25%–295.67%. Additionally, MOSY PHYCOLLREPAIR enhanced wound healing by 8.33%–19.44% at concentrations between 5 and 100 μg/mL, provided 39.36% to 70.26% skin barrier protection against chemical peel at 100 to 500 μg/mL, and offered 20.15%–68.45% UVB irradiation protection at 200 to 2000 μg/mL.

**Conclusion:**

This study marks the first application of fermented blank bee honey as an active cosmetic ingredient. The findings conclusively establish MOSY PHYCOLLREPAIR as a safe and highly effective cosmetic component, delivering a broad range of anti‐aging and skin health benefits.

## 1 Introduction

Honey, a natural product with a long‐standing reputation for its nutritional and medicinal benefits, is a complex mixture of over 181 compounds. These include a variety of bioactive substances such as flavonoids, phenolic acids, organic acids, enzymes, vitamins, and minerals [1]. The bioactivity of honey is influenced by factors such as geographical origin, climate, and the cultivation season. Research indicates that dark honeys generally provide more health benefits than light honeys, and multifloral honeys are often considered more beneficial than monofloral ones [2]. Honey is particularly noted for its antioxidant and antimicrobial properties [3, 4, 5, 6]. Historically, it has been used to treat a range of dermatological conditions, including infections, atopic dermatitis, psoriasis, necrotizing fasciitis, ulcers, and thermal injuries [7, 8, 9, 10, 11, 12, 13]. In the cosmetic industry, honey has demonstrated excellent moisturizing and anti‐wrinkle effects [9, 11, 14, 15]. A 4‐week clinical study on hand creams containing varying percentages of multifloral honey showed significant improvements in skin moisturization (up to 29.7%), smoothness (up to 21.3%), wrinkle area reduction (up to 21.4%), and mean wrinkle depth reduction (up to 11.7%), with the best skin barrier function observed in creams containing 5% and 10% honey [15].

Xinjiang Nilka black bee honey, produced in Nilka County, Xinjiang province, China, originates from an area rich in 276 nectar‐producing plants, including 76 medicinal species such as *Vicia cracca* (Cow vetch), *Thymus vulgaris* (Thyme), *Elsholtzia ciliata* (Mosquito plant), *Solidago canadensis* (Canada goldenrod), *Cirsium japonicum* (Japanese thistle), *Arctium lappa* (Burdock), *Codonopsis pilosula* (Dangshen), *Mentha haplocalyx* (Field mint), *Artemisia argyi* (Mugwort), Fritillaria (Beimother), and *Nepeta cataria* (Catmint). These plants bloom from May to September, yielding approximately 280 tons of honey annually [16]. Characterized by high glucose and fructose content (over 70%), low sucrose (≤ 1.24%), and low water content (≤ 18.5%), Nilka black bee honey also exhibits and is rich in vitamin E, vitamin B2, vitamin B6, nicotinic acid, pantothenic acid, and folic acid. It also features a high potassium‐to‐sodium ratio, high calcium content, and is rich in selenium and amino acids [16, 17]. Since 2016, efforts have been made to incorporate Nilka black bee honey into cosmetics and skincare products.

Despite honey's efficacy as a natural cosmetic ingredient, formulating stable and sensory‐pleasing honey‐containing cosmetics presents challenges. The sticky residue left after water evaporation from honey‐based cosmetics can be unpleasant for consumers [15]. To address this, fermentation technology was used to transform granulated solid honey into a clear, amber‐colored liquid named MOSY PHYCOLLREPAIR. Fermentation enhances biological activities and improves product compatibility by converting high‐molecular‐weight compounds into lower‐molecular‐weight structures, a technique well‐established in cosmetic ingredient development [18, 19].

Zebrafish have become a valuable model in dermatological research and cosmetic evaluation due to their genetic similarity to humans (up to 82% in disease‐related genes) and their widespread use in toxicity testing, disease modeling, drug discovery, and efficacy evaluations [20, 21, 22, 23, 24, 25, 26, 27, 28, 29, 30, 31]). In this study, zebrafish were used to assess the safety and potential skincare benefits of MOSY PHYCOLLREPAIR.

## 2 Materials and Methods

### 2.1 Chemicals and Reagents

**FIGURE 1:**
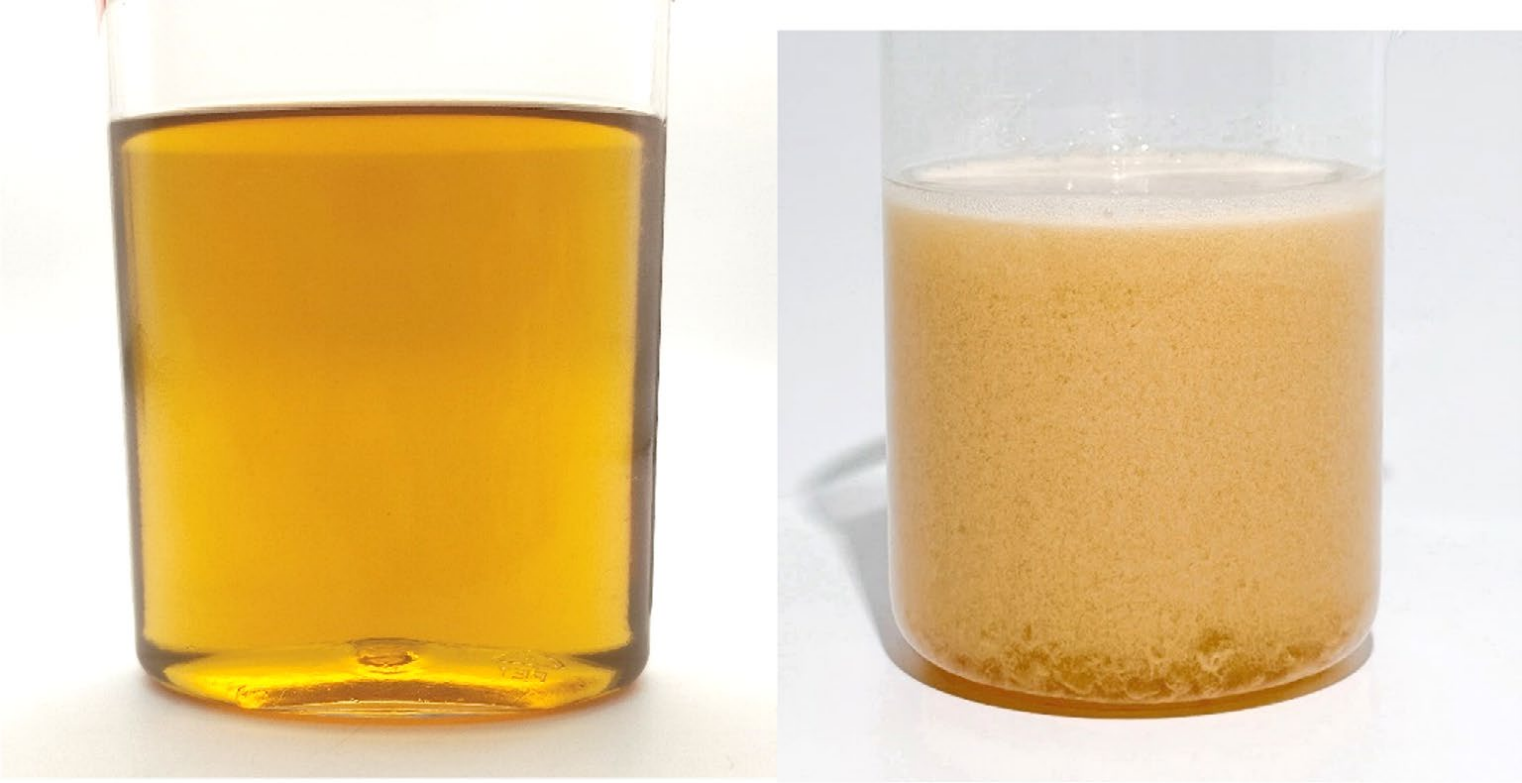
Comparative Visualization of MOSY PHYCOLLREPAIR and Nilka black bee honey. Left: MOSY PHYCOLLREPAIR. Right: Nilka black bee honey (Table 1).

MOSY PHYCOLLREPAIR was produced by fermenting Xinjiang black bee honey with bifidobacterium and subsequently refining the fermentation product using low‐temperature microjet extraction technology. This fermentation process transformed the granulated solid honey into a clear, amber‐colored liquid, as depicted in Figure 1.

**TABLE 1:** Primer sequences for qRT‐PCR.

Name of the gene	Primer sequences
*Col1a1a*	F: 5′‐TAGCCCCTATGGACGTTGGT‐3′, R: 5′‐CGCAGGTCTAAGCAAGTGGA‐3′
*Col1a1b*	F: 5′‐TGGCATGACCGGCCCTATTG‐3′, R: 5′‐CTCTCCTTTAGCACCAGGCTGT‐3′
*Col1a2*	F: 5′‐GAGGCCAGCCTGGTAACATT‐3′, R: 5′‐GTTACCATCAGGACCAGGGC‐3′
*Elna*	F: 5′‐AAAACCAGGTTACGGCTCTGT‐3′, R: 5′‐TCCTCCTGGATAAGCTCCGTATC‐3′
*Sirt1*	F: 5′‐CAACAACACTAGACCGACTGAAC‐3′, R: 5′‐GCTGCTGAATGAACCGATAGG‐3′
*β‐Actin*	F: 5′‐GCTGACAGGATGCAGAAGGA‐3′, R: 5′‐TAGAAGCATTTGCGGTGGAC‐3′

The following reagents were utilized in this study: *Rehmannia glutinosa* Libosch extract (batch number: N103002586), procured from Aladin Group; 3,4‐dichloroaniline (DCA), caffeine, glutathione, indomethacin, CuSO_4_, tricaine, Sudan black B, methanol, glucose, ectoin, and lauryl sodium sulfate (SLS), all sourced from Sigma‐Aldrich; 4% paraformaldehyde tissue fixative from Biosharp; H_2_DCFDA from Thermo Fisher; and RNAsimple total RNA kit (Catalog number: DP419) from Tiangen. Additionally, ergothioneine with a purity of 99.99% was obtained from GeneIII Bio‐Tec Co Ltd.

### 2.2 Zebrafish Maintenance and Embryo Collection

Adult wild‐type zebrafish ( *Danio rerio* ) of the AB strain were sourced from the Zebrafish International Resource Center (ZIRC; University of Oregon, Eugene, OR, USA). The husbandry of the fish colony and the procedure for embryo collection were executed according to established methods [29]. Animal‐related operations were approved by the Department of Health, Hong Kong, SAR, China 23–90 (No. 23–86 in DH/HT&A/8/218 Pt524.10.2023). Briefly, the adult zebrafish were maintained under a controlled 14‐hour light/8‐hour dark photoperiod and were fed three daily meals of freshly hatched brine shrimp. During embryo collection, embryos were harvested from a minimum of ten breeding pairs per batch, ensuring that only robust and healthy embryos were selected for subsequent experimentation. The chosen embryos were nurtured in E3 medium, a zebrafish‐specific culture medium composed of 290 mg/L NaCl, 13.3 mg/L KCl, 48.3 mg/L CaCl_2_·2H_2_O, and 81.5 mg/L MgCl_2_·6H_2_O, with the pH carefully adjusted to 7.2. Throughout the study, the health and viability of the embryos were rigorously monitored, with only the healthiest specimens being employed for experimental analyses.

### 2.3 Zebrafish Embryo Toxicity Analysis

Building on the methodology of Xu et al. [29], this study assessed the toxicity of MOSY PHYCOLLREPAIR on zebrafish embryos at the 4‐ to 128‐cell stage. Embryos were exposed individually to MOSY PHYCOLLREPAIR solutions at concentrations of 0.29, 0.64, 1.40, 3.08, and 6.67 mg/mL in 96‐well plates, with each well containing a single embryo and 20 wells dedicated to each concentration. Untreated embryos served as negative controls, while those treated with 0.004 mg/mL dexamethasone (Dex) were positive controls. The exposure period lasted until 48 h post‐fertilization (hpf). Embryos were classified as dead if they exhibited coagulation, absence of heartbeat, or failure to detach the tail from the yolk. Any phenotypic abnormalities diverging from the typical developmental progression were meticulously documented.

### 2.4 Antioxidation Assay

This experiment was carried out following the protocol established by Han et al. [28]. Zebrafish embryos at 48 h post‐fertilization (hpf) were subjected to MOSY PHYCOLLREPAIR solutions at concentrations of 25, 50, 150, 250, and 500 μg/mL. The exposure was conducted in 96‐well plates, with each well containing a single embryo and 24 wells designated for each treatment group. Untreated embryos served as negative controls, while embryos treated with 0.1 mg/mL glutathione (GSH) were used as positive controls. After a 24‐h exposure period, embryos from each group were live‐stained with 10 μM H₂DCFDA for 2 h to visualize reactive oxygen species (ROS). Subsequently, the embryos were anesthetized with 0.014% tricaine and imaged using a fluorescence microscope (Nikon DS‐Qi2) under uniform settings for all samples. The green fluorescence signal, indicative of ROS, was quantified using ImageJ software. Anti‐ROS efficacy was calculated as $\frac{{\text{Signal}}_{\_\text{negative}}-{\text{Signal}}_{\_\text{sample}}}{{\text{Signal}}_{\_\text{negative}}}\times 100\%$.

### 2.5 Anti‐Inflammation Assay

At 72 hpf, zebrafish embryos were treated with 10 μM CuSO₄ to induce inflammation and subsequently exposed to MOSY PHYCOLLREPAIR at concentrations of 25, 50, 150, 250, and 500 μg/mL. Each treatment group consisted of 24 embryos. The experimental design incorporated three control groups: negative controls (untreated, normal embryos), model controls (embryos treated solely with 10 μM CuSO₄), and positive controls (embryos treated with both 10 μM CuSO₄ and 10 μM indomethacin, a known anti‐inflammatory agent). After a 40‐minute exposure, the embryos were fixed using a 4% paraformaldehyde tissue fixative and stained with 0.04% Sudan black B for 1 hour. These procedures followed the established methods by Xu et al. [29] and Han et al. [28]. Finally, the number of neutrophils in the midline region of each embryo was quantified to assess the inflammatory response. Anti‐inflammation efficacy was calculated as $\frac{\text{Number}\;{\text{of neutrophil}}_{\_\text{model}}-\text{Number}\;{\text{of neutrophil}}_{\_\text{sample}}}{\text{Number}\;{\text{of neutrophil}}_{\_\text{model}}-\text{Number}\;{\text{of neutrophil}}_{\_\text{negative}}}\times 100\%$.

### 2.6 Type I Collagen (col1a1a, col1a1b, and col1a2), Elastin Gene (Elna), and sirt1 Gene Expression Assay

Zebrafish embryos at 120 hpf were subjected to MOSY PHYCOLLREPAIR solutions across a range of concentrations: 25, 50, 150, 250, and 500 μg/mL. The exposure setup involved 24‐well plates, with each well containing 12 embryos in 2.5 mL of solution, and three replicate wells for each concentration. The experimental design incorporated negative controls, comprising untreated, normal embryos, and positive controls, which were embryos treated with 50 μg/mL glutathione (GSH). Following a 24‐hour exposure period, embryos from each replicate well were pooled for subsequent analysis. Quantitative reverse transcription polymerase chain reaction (qRT‐PCR) was employed to assess the expression levels of type I collagen genes (*col1a1a*, *col1a1b*, and *col1a2*), the elastin gene (*elna*), and the *sirt1* gene, although the corresponding primer sequences for these genes are not provided in this excerpt.

### 2.7 ATP Assay

Zebrafish embryos at 120 hpf were treated with MOSY PHYCOLLREPAIR at concentrations of 25, 50, 150, 250, and 500 μg/mL. The exposure setup was arranged in 24‐well plates, with 12 embryos per well in 2.5 mL of solution, and three replicate wells for each concentration. The experimental design incorporated negative controls, which were untreated normal embryos, and positive controls, which were embryos treated with 4% glucose. After a 6‐hour exposure period, 10 embryos from each replicate well were randomly selected and pooled. These embryos were then anesthetized using 0.02% tricaine, homogenized in 120 μL of lysis solution (S0026, ATP Assay Kit, Beyotime Biotechnology, China) on ice, and centrifuged at 12,000 g at 4°C for 5 minutes. The supernatant was collected and used for ATP quantification following the technical instructions provided with the ATP Assay Kit.

### 2.8 Tail Fin Regeneration Assay

Adhering to the protocols outlined by Xu et al. [29] and Han et al. [28], this experiment involved zebrafish embryos at 3 days post‐fertilization (dpf). The embryos were first anesthetized with a 0.02% tricaine solution to ensure humane handling, and their tail fins were precisely amputated using a sharp blade. Following amputation, the embryos were exposed individually to MOSY PHYCOLLREPAIR solutions across a range of concentrations: 5, 10, 30, 50, and 100 μg/mL. The exposure was conducted in 96‐well plates, with each well containing a single embryo, and 24 wells designated for each treatment group. The experimental design incorporated several control groups: negative controls consisting of non‐amputated, normal embryos; model controls comprising amputated embryos without any treatment; and positive controls featuring amputated embryos treated with 1 mg/mL *Rehmannia glutinosa* Libosch extract, a compound known to promote regeneration. After a 48‐hour exposure period, the regenerated tail fins of the embryos were photographed using a stereomicroscope (MZ10F, Leica). The relative length of the regenerated tail fins was then measured using ImageJ software to quantify the extent of regeneration. Regeneration enhancing rate was calculated as $\frac{{\text{Length}}_{\_\text{sample}}-{\text{Length}}_{\_\text{model}}}{{\text{Length}}_{\_\text{negative}}-{\text{Length}}_{\_\text{model}}}\times 100\%$.

### 2.9 Skin Barrier Protection Assay

At 48 hpf, zebrafish embryos were subjected to MOSY PHYCOLLREPAIR at concentrations of 100, 200, 600, 1000, and 2000 μg/mL. Each treatment was administered in 6‐well plates, containing 20 embryos in 4 mL of solution per well. To each well, lactic acid (1.1 mg/mL) was added as a chemical peel reagent, and fluorescein sodium (0.5 mg/mL) was included as a fluorescence dye. The experimental design incorporated three sets of controls: negative controls, which were embryos treated with only 0.5 mg/mL fluorescein sodium; model controls, which included embryos treated with both 1.1 mg/mL lactic acid and 0.5 mg/mL fluorescein sodium; and positive controls, which comprised embryos treated with 150 μg/mL ectoin, 1.1 mg/mL lactic acid, and 0.5 mg/mL fluorescein sodium. After a 2‐hour exposure, the embryos were rinsed with water, anesthetized using a 0.02% tricaine solution, and then imaged using a fluorescence microscope (Nikon DS‐Qi2). The fluorescence signal in the tail region of the embryos, indicative of skin barrier integrity, was quantified using ImageJ software. The skin barrier protection rate was calculated as $\frac{{\text{Transluence}}_{\_\text{sample}}-{\text{Transluence}}_{\_\text{model}}\;}{{\text{Transluence}}_{\_\text{negative}}-{\text{Transluence}}_{\_\text{model}}}\times 100\%$.

### 2.10 UV Irradiation Protection Assay

Zebrafish embryos at 72 hpf were treated with MOSY PHYCOLLREPAIR at concentrations of 100, 200, 600, 1000, and 2000 μg/mL. Each treatment group consisted of 24 embryos placed in 6 mL of solution within a 6‐cm diameter Petri dish. Following a 1‐hour exposure period, the embryos were anesthetized with a 0.02% tricaine solution, gently rinsed, and returned to their respective solutions. Subsequently, the embryos were subjected to 0.3 J/cm^2^ of UVB irradiation and then incubated for an additional 24 hours. After incubation, the embryos were anesthetized once more with 0.02% tricaine and photographed using a stereomicroscope (MZ10F, Leica). The tail area of each embryo was quantified using ImageJ software to assess the extent of UV damage. The experimental design incorporated several control groups: negative controls, which were untreated normal embryos; model controls, which were embryos irradiated with UVB only; and positive controls, which were embryos treated with 5 mg/mL ergothioneine and exposed to UVB, serving as a benchmark for UV protection. The UV protection rate was calculated as $\frac{{\text{Area}}_{\_\text{sample}}-{\text{Area}}_{\_\text{model}}}{{\text{Area}}_{\_\text{negative}}-{\text{Area}}_{\_\text{model}}}\times 100\%$.

### 2.11 Melanin Suppression Assay

Adhering to the methodology detailed by Han et al. [28], this experiment assessed the melanin suppression efficacy of MOSY PHYCOLLREPAIR. Zebrafish embryos at the 6 to 8 hpf stage were individually exposed to MOSY PHYCOLLREPAIR solutions at concentrations ranging from 25 to 500 μg/mL. Each embryo was placed in 0.2 mL of solution within a well of a 96‐well plate, with 20 wells dedicated to each treatment concentration. The experimental design incorporated several control groups: a 100% whitening efficacy control using 30 μg/mL phenylthiourea (PTU), a positive control with 5 mg/mL kojic acid, and untreated normal embryos serving as negative controls. Following exposure until 48 hpf, the embryos were anesthetized with 0.014% tricaine to facilitate imaging. They were then photographed using a microscope (Nikon DS‐Qi2) with uniform settings across all samples to ensure consistency. The transmittance signal in the head and body regions of the embryos, indicative of melanin content, was quantified using ImageJ software. The melanin suppression rate was calculated as $\frac{{\text{Transluence}}_{\_\text{negative}}-{\text{Transluence}}_{\_\text{sample}}}{{\text{Transluence}}_{\_\text{negative}}-{\text{Transluence}}_{\_\mathrm{PTU}}}\times 100\%$.

### 2.12 Statistical Analysis

Excel was used to analyze the data with the results expressed as Mean ± SD. The comparisons between groups were performed using two‐tailed *t*‐test and Wilcoxon signed‐rank tests, with a *p* < 0.05 considered as a significant difference.

## 3 Results

### 3.1 MOSY PHYCOLLREPAIR Exhibited Low Toxicity to Zebrafish Embryos

**FIGURE 2:**
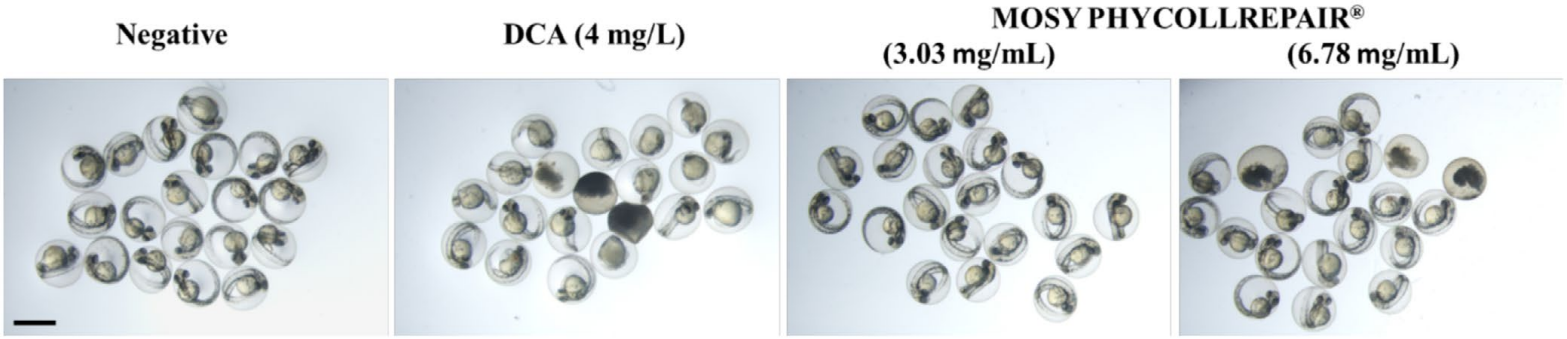
MOSY PHYCOLLREPAIR exhibited low toxicity to zebrafish embryos. Scale bar = 0.5 mm.

The findings from the zebrafish embryo developmental toxicity assay, as depicted in Figure 2, indicated that embryos in the negative control group developed without any observed adverse effects. Conversely, the positive control group, which was treated with 4 mg/L dexamethasone (Dex), displayed a mortality rate of 35% and a malformation rate of 90%. In the case of MOSY PHYCOLLREPAIR, at the highest tested concentration of 6.78 mg/mL, the observed mortality rate was 15%, and 15% of the embryos developed yolk edema. Notably, no signs of developmental toxicity were detected at concentrations of 3.08 mg/mL or lower. These results suggest that MOSY PHYCOLLREPAIR poses a low risk of toxicity to zebrafish embryos at concentrations up to 3.08 mg/mL, indicating its potential safety for dermatological applications.

### 3.2 Antioxidant Activity of MOSY PHYCOLLREPAIR in Zebrafish Embryos

**FIGURE 3:**
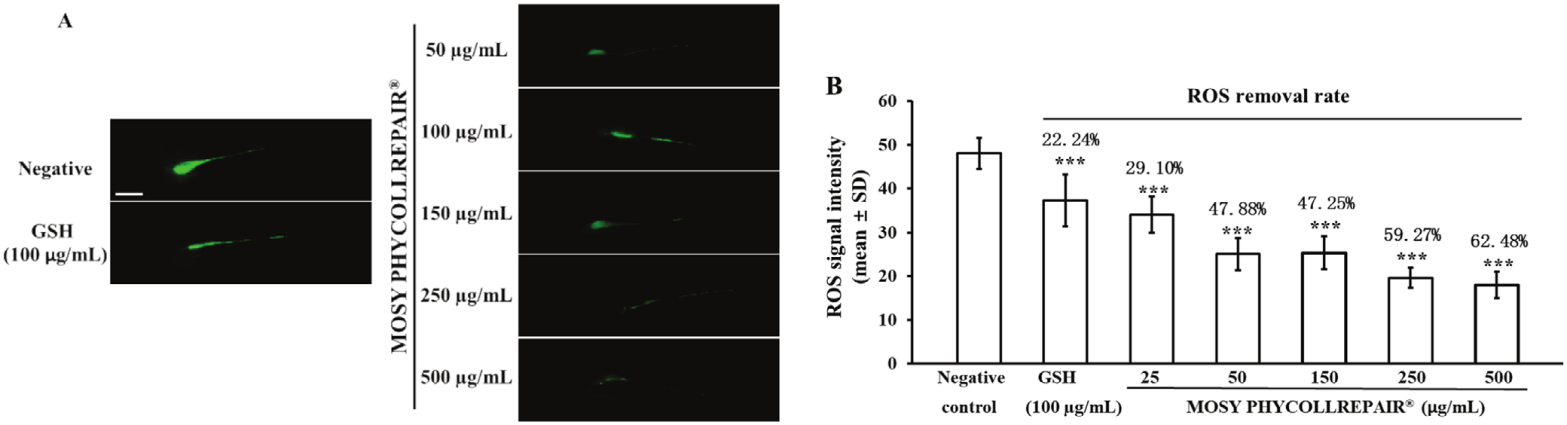
MOSY PHYCOLLREPAIR exhibited a dose‐dependent reduction in ROS levels within zebrafish embryos. Scale bar = 100 μm. Asterisks denote statistical significance compared to the negative control: ***p < 0.001.

The antioxidant assays, illustrated in Figure 3A, show that treatments with both glutathione (GSH) at 100 μg/mL and various concentrations of MOSY PHYCOLLREPAIR significantly diminished the reactive oxygen species (ROS) fluorescence signal when compared to the negative control group. A quantitative assessment of the ROS signal intensity, presented in Figure 3B, indicated that GSH reduced the ROS signal from a baseline of 48.00 to 37.33, marking a 22.24% decrease. In contrast, MOSY PHYCOLLREPAIR at concentrations of 25, 50, 150, 250, and 500 μg/mL led to more pronounced reductions in the ROS signal, achieving decreases of 29.10%, 47.88%, 47.25%, 59.27%, and 62.48%, corresponding to ROS signal values of 34.03, 25.02, 25.32, 19.60, and 18.01, respectively. These findings underscore the potent antioxidant capabilities of MOSY PHYCOLLREPAIR, with its efficacy appearing to be concentration‐dependent.

### 3.3 Anti‐Inflammation Activity of MOSY PHYCOLLREPAIR in Zebrafish Embryos

**FIGURE 4:**
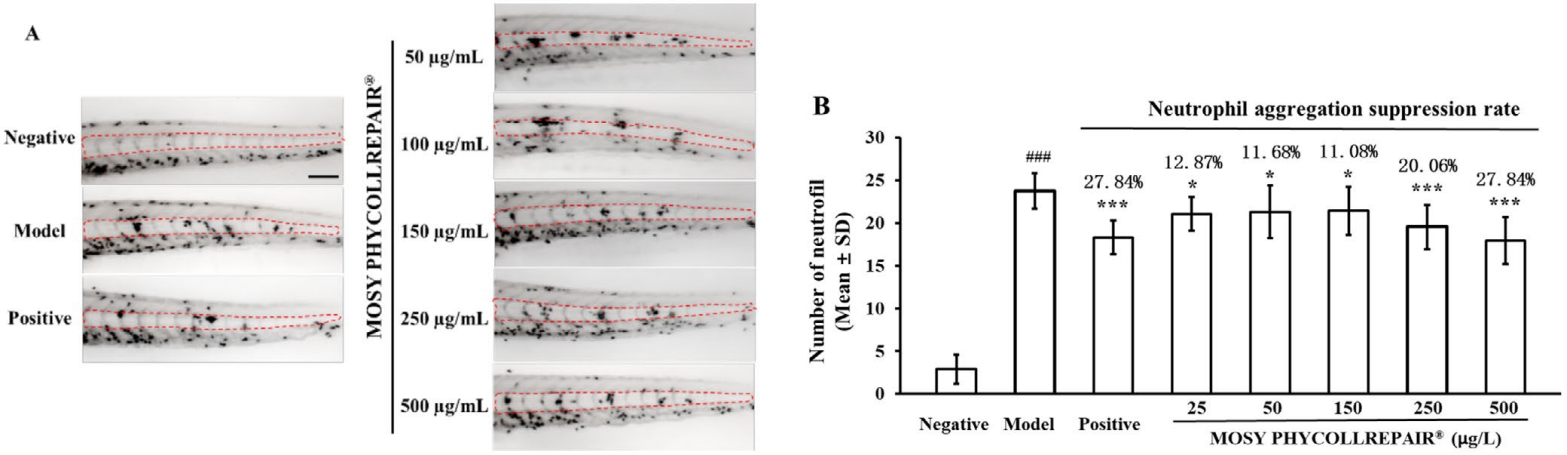
MOSY PHYCOLLREPAIR exhibited anti‐inflammation activity in zebrafish embryos. Model: 10 μM CuSO_4_ treatment. Positive control: 10 μM indomethacin and 10 μM CuSO_4_ cotreatment. Scale bar = 50 μm. Hash symbols (#) denote significance compared to the negative control: ### *p* < 0.001. Asterisks (*) denote significance compared to the model control: *p < 0.05, ***p < 0.001.

The anti‐inflammatory assays, depicted in Figure 4A, illustrate that while the negative control group displayed minimal neutrophil accumulation in the midline region, the model control group treated with 10 μM CuSO₄ exhibited a significant increase in neutrophil aggregation. Conversely, treatments with 10 μM indomethacin (positive control) and MOSY PHYCOLLREPAIR at concentrations of 25, 50, 150, 250, and 500 μg/mL all led to a noticeable reduction in neutrophil accumulation compared to the model control. Quantitative analysis of neutrophil numbers, as shown in Figure 4B, indicated an average of 2.88 neutrophils in normal embryos and a substantial increase to 23.75 neutrophils in embryos treated with 10 μM CuSO₄. The positive control treatment with indomethacin reduced neutrophil aggregation by 27.82%, resulting in 18.31 neutrophils. MOSY PHYCOLLREPAIR treatments further diminished neutrophil aggregation, with reductions ranging from 12.87% to 27.84% across the tested concentrations, corresponding to 21.06 to 17.94 neutrophils, respectively. Statistical analysis confirmed that all tested concentrations of MOSY PHYCOLLREPAIR significantly (*p* < 0.05) suppressed neutrophil aggregation, demonstrating its robust anti‐inflammatory activity.

### 3.4 MOSY PHYCOLLREPAIR Enhanced Expression of Type I Collagen, Elastin, and sirt1 Genes in Zebrafish Embryos

**FIGURE 5:**
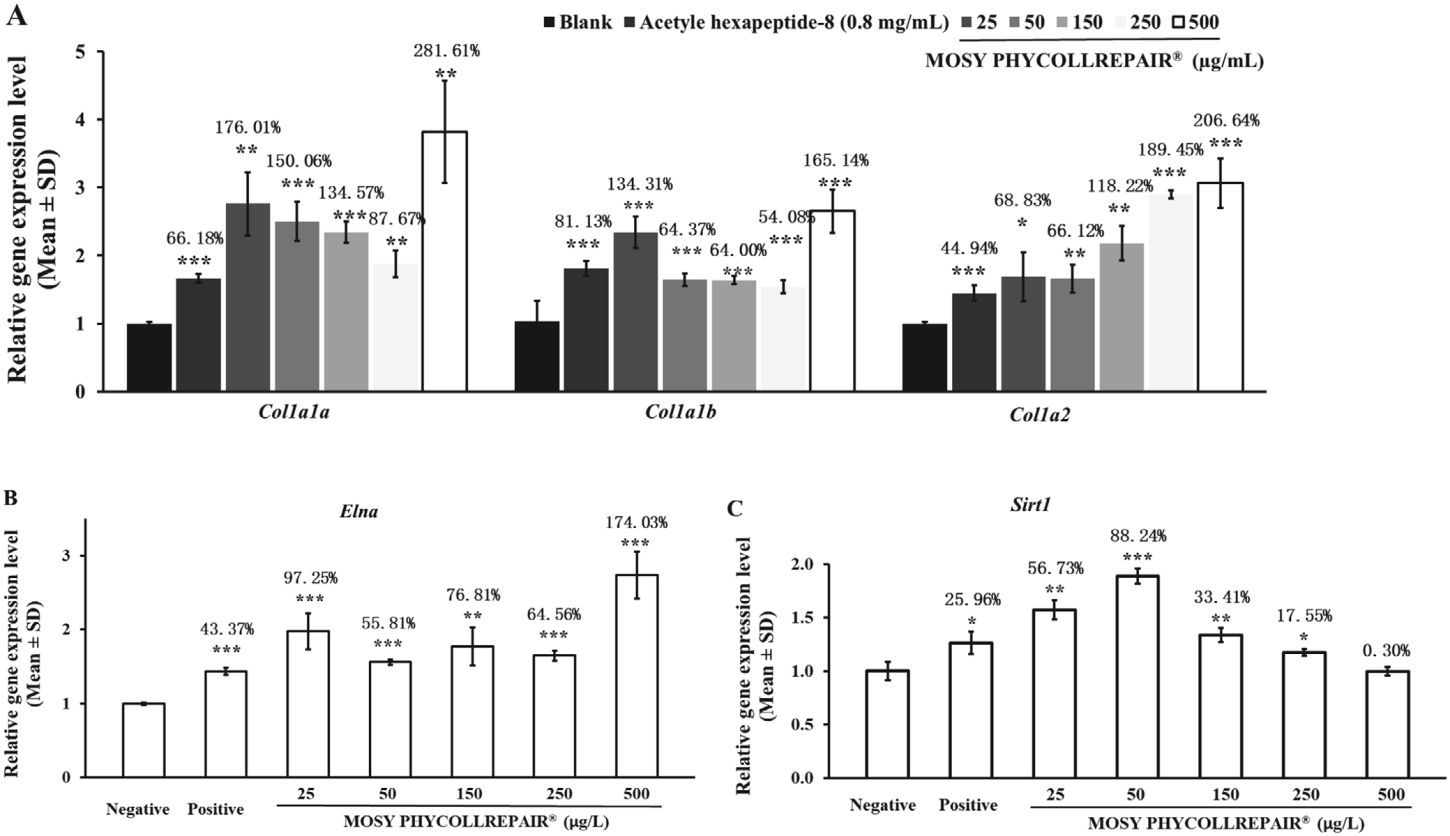
MOSY PHYCOLLREPAIR upregulated the expression of type I collagen (*col1a1a*, *col1a1b*, and *col1a2*), elastin (*elna*), and *sirt1* genes in zebrafish embryos. Positive control: 0.8 mg/mL GSH. Asterisks denote statistical significance compared to the negative control: *p < 0.05; **p < 0.01; ***p < 0.001.

Results from the collagen type I gene regulation experiment (Figure 5A) demonstrated that, compared to the negative control, 0.8 mg/mL GSH significantly upregulated the expression of *col1a1a*, *col1a1b*, and *col1a2* by 66.18% (*p* = 0.0001), 81.13% (*p* = 0.0003), and 44.94% (*p* = 0.0028), respectively. MOSY PHYCOLLREPAIR, at the tested concentrations of 25, 50, 150, 250, and 500 μg/mL, also significantly (*p* < 0.05) upregulated the expression of these genes. The increases in expression ranged from 87.67% to 281.61% for *col1a1a*, 64.00% to 165.14% for *col1a1b*, and 66.12% to 206.64% for *col1a2*. These findings highlight the potent collagen‐regenerating properties of MOSY PHYCOLLREPAIR, particularly at lower concentrations.

In the elastin gene regulation experiment (Figure 5B), 0.8 mg/mL GSH significantly increased the expression of the *elna* gene by 43.43% (*p* = 0.0008) compared to the negative control. MOSY PHYCOLLREPAIR treatments at 25, 50, 150, 250, and 500 μg/mL also significantly upregulated *elna* gene expression by 97.25% (*p* = 0.0026), 55.81% (*p* = 0.0002), 76.81% (*p* = 0.0070), and 174.03% (*p* = 0.0007), respectively. These results indicate that MOSY PHYCOLLREPAIR effectively enhances elastin regeneration, further supporting its potential as a powerful anti‐aging agent.

The results of the *sirt1* gene expression analysis, as illustrated in Figure 5C, indicate that, compared to the negative control, treatment with 80 μg/mL glutathione (GSH), serving as the positive control, significantly elevated the expression of the sirt1 gene by 25.96% (*p* = 0.02). When zebrafish embryos were treated with MOSY PHYCOLLREPAIR at concentrations of 25, 50, 150, 250, and 500 μg/mL, the expression of the SIRT1 gene was increased by 56.73% (*p* = 0.0014), 88.24% (*p* = 0.0002), 33.41% (*p* = 0.0058), 17.55% (*p* = 0.030), and 0.30% (*p* = 0.096), respectively. These findings suggest that MOSY PHYCOLLREPAIR has considerable anti‐aging potential, particularly at lower concentrations. The most significant enhancement in sirt1 gene expression was observed at a concentration of 50 μg/mL, highlighting the compound's ability to activate genes associated with longevity and anti‐aging processes.

### 3.5 Enhancement of ATP Level by MOSY PHYCOLLREPAIR in Zebrafish Embryos

**FIGURE 6:**
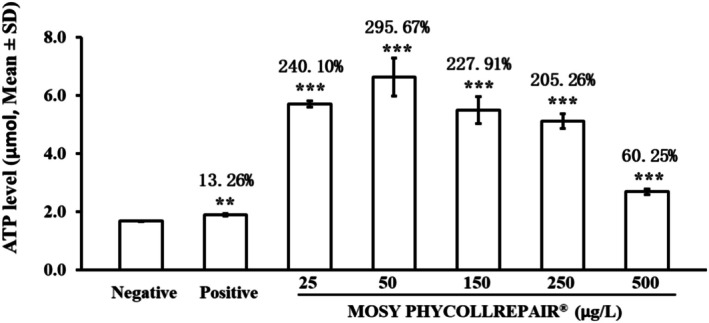
MOSY PHYCOLLREPAIR enhanced ATP levels in zebrafish embryos. Positive: 4% glucose. Asterisks (***) denote significance compared to the model control: **p < 0.01; ***p < 0.001.

The ATP analysis, as depicted in Figure 6, revealed significant differences in ATP levels among the treatment groups compared to normal zebrafish embryos, which had a baseline ATP level of 1.67 μmol. Treatment with 4% glucose notably increased ATP levels to 1.90 μmol, marking a 13.26% increase (*p* = 0.0023). Furthermore, MOSY PHYCOLLREPAIR at concentrations of 25, 50, 150, 250, and 500 μg/mL significantly elevated ATP levels to 5.70 μmol (240.10% increase), 6.63 μmol (295.67% increase), 5.50 μmol (227.91% increase), 5.12 μmol (205.27% increase), and 2.69 μmol (60.25% increase), respectively (*p* < 0.001 for all concentrations). These findings demonstrate that MOSY PHYCOLLREPAIR can substantially enhance mitochondrial function, leading to more efficient ATP production.

### 3.6 MOSY PHYCOLLREPAIR Enhanced Wound Healing in Zebrafish Embryos

**FIGURE 7:**
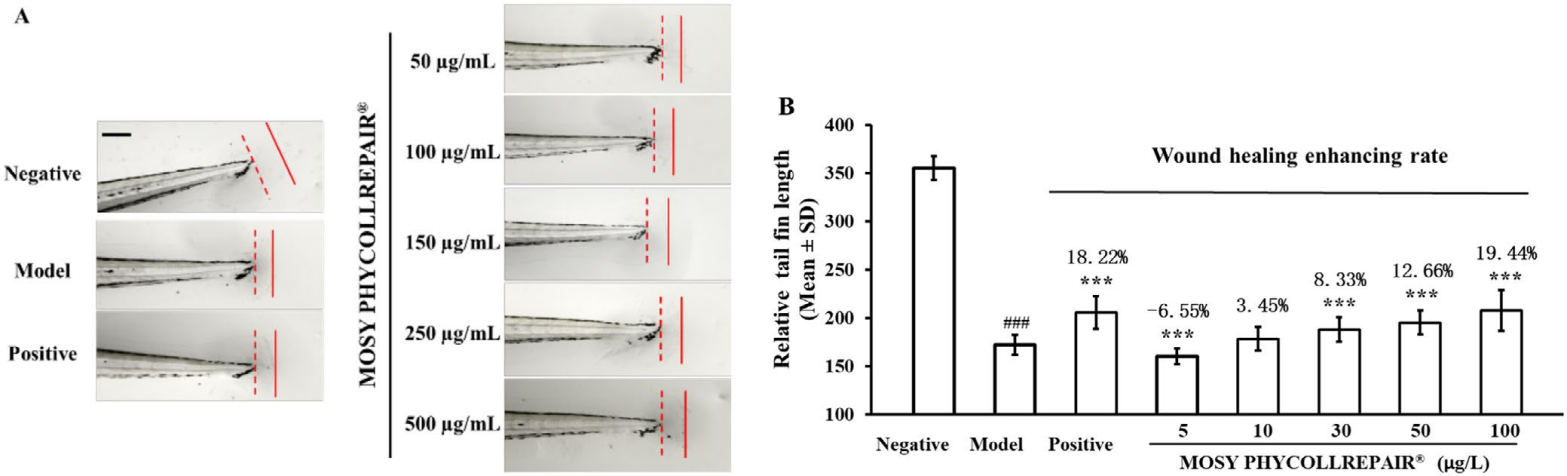
MOSY PHYCOLLREPAIR inhibited melanin synthesis in zebrafish embryos. Scale bar = 50 μm. Hash symbols (###) denote significance compared to the negative control: *P* < 0.001; asterisks (***) denote significance compared to the model control: ***p < 0.001.

The wound healing experiment, as shown in Figure 7A, presents significant tail fin regeneration in zebrafish embryos treated with 1 mg/mL *Rehmannia glutinosa* Libosch extract and MOSY PHYCOLLREPAIR at concentrations of 150, 250, and 500 μg/mL, compared to the model control group. The quantitative measurement results depicted in Figure 7B reveal the relative tail fin lengths as follows: 355.26 for non‐amputated embryos (negative control), 172.29 for amputated embryos (model control), and 205.62 for the positive control group, which represents an 18.22% increase (*p* < 0.12). The MOSY PHYCOLLREPAIR treatments at concentrations of 25, 50, 150, 250, and 500 μg/mL resulted in relative tail fin lengths of 160.31 (6.55% decrease, *p* < 0.001), 178.60 (3.45% increase, *p* = 0.001), 188.44 (8.83% increase, *p* < 0.001), 195.46 (12.66% increase, *p* < 0.001), and 207.86 (19.44% increase, *p* < 0.001), respectively. These findings indicate that MOSY PHYCOLLREPAIR exhibits a positive, dose‐dependent enhancement of wound healing in damaged skin.

### 3.7 MOSY PHYCOLLREPAIR Strengthened Skin Barrier Integrity in Zebrafish Embryos

**FIGURE 8:**
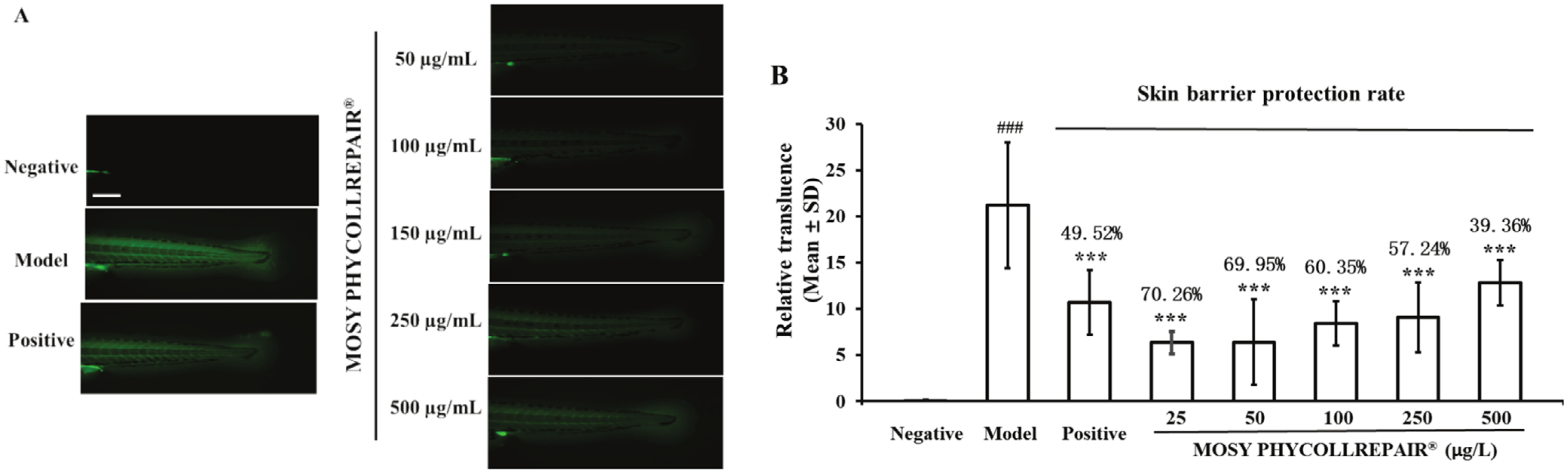
MOSY PHYCOLLREPAIR ameliorated skin barrier damage induced by acetic acid. Model: 1.1 mg/mL lactic acid. Positive control: 150 μg/mL ectoin co‐treated with 1.1 mg/mL lactic acid. Scale bar = 50 μm. Hash symbols (###) denote significance compared to the negative control: *P* < 0.001; asterisks (***) denote significance compared to the model control: *P* < 0.001.

The skin barrier protection experiment, illustrated in Figure 8A, shows that fluorescein sodium (which exhibits green fluorescence) did not permeate the skin of normal embryos (negative control). However, significant penetration was observed in the presence of 1.1 mg/mL lactic acid (model control), indicating compromised skin barrier integrity. In contrast, embryos treated with 150 μg/mL ectoin and various concentrations of MOSY PHYCOLLREPAIR displayed reduced green fluorescence, suggesting improved skin barrier integrity.

Quantitative analysis of fluorescence intensity in the tail area, as shown in Figure 8B, yielded the following values: 0.081 for normal embryos (negative control) and 21.18 for the model control. Compared to the model control, fluorescence intensity was significantly reduced with 150 μg/mL ectoin treatment to 10.73 (49.52% decrease, *p* < 0.001), and with MOSY PHYCOLLREPAIR treatments at concentrations of 25, 50, 150, 250, and 500 μg/mL to 6.36 (70.26% decrease, *p* = 0.078), 6.42 (69.95% decrease, *p* < 0.001), 8.45 (60.35% decrease, *p* < 0.001), 9.10 (57.24% decrease, *p* < 0.001), and 12.88 (39.36% decrease, *p* < 0.001), respectively. These results demonstrate that MOSY PHYCOLLREPAIR significantly diminished the fluorescence signal of permeated fluorescein sodium in the skin, thereby enhancing skin barrier integrity in a dose‐dependent manner. This underscores the robust skin barrier protective properties of MOSY PHYCOLLREPAIR.

### 3.8 MOSY PHYCOLLREPAIR Mitigated UV Irradiation‐Induced Damage in Zebrafish Embryos

**FIGURE 9:**
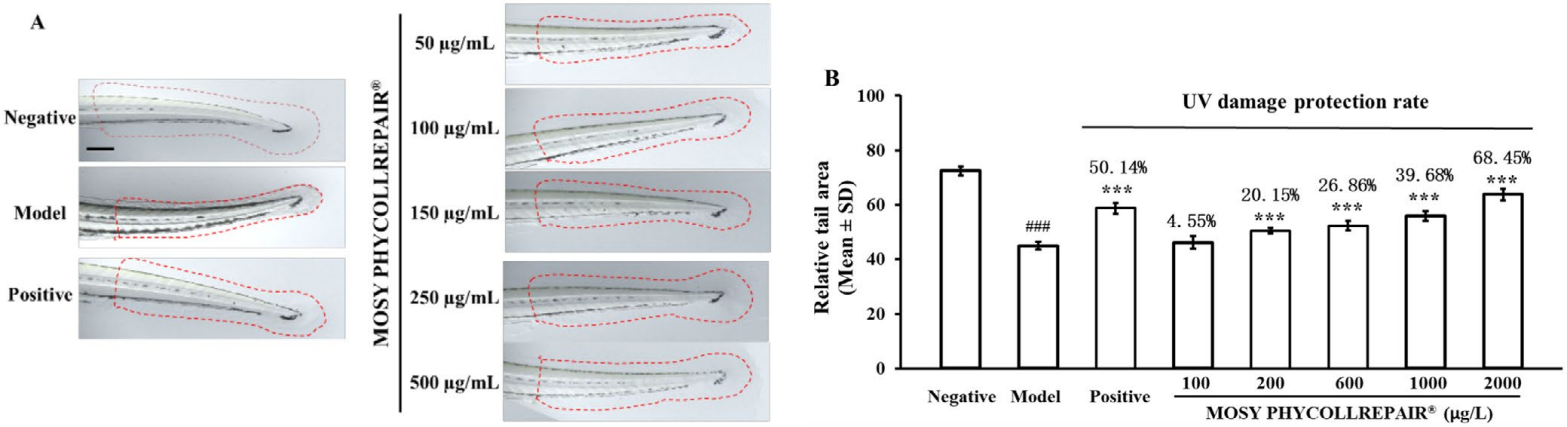
MOSY PHYCOLLREPAIR alleviated UVB irradiation‐induced damage in zebrafish embryos. Model: 0.3 J/cm^2^ UVB irradiation. Positive control: 5 mg/mL ergothioneine and 0.3 J/cm^2^ UVB irradiation. Scale bar = 50 μm. Hash symbols (###) denote significance compared to the negative control: *P* < 0.001; asterisks (***) denote significance compared to the model control: *P* < 0.001.

The UV irradiation protection experiment, as depicted in Figure 9A, demonstrates that while normal embryos (negative control) showed no signs of damage, exposure to 0.3 J/cm^2^ UVB irradiation led to significant damage in zebrafish embryos, characterized by embryo shrinkage. Treatment with 5 mg/mL ergothioneine and MOSY PHYCOLLREPAIR at concentrations of 100, 200, 600, 1000, and 2000 μg/mL significantly reduced the shrinkage caused by UVB irradiation.

Quantitative analysis of the relative tail region areas, presented in Figure 9B, yielded the following results: 72.45 for normal embryos (negative control), and 44.91 for the model control group (UVB irradiated). Ergothioneine treatment at 5 mg/mL resulted in a relative tail area of 58.72, indicating a 50.14% alleviation of damage (*p* < 0.001). MOSY PHYCOLLREPAIR treatments at 100, 200, 600, 1000, and 2000 μg/mL showed relative tail areas of 46.16 (4.55% increase, *p* = 0.078), 50.46 (20.15% alleviation, *p* < 0.001), 52.31 (26.86% alleviation, *p* < 0.001), 55.84 (39.68% alleviation, *p* < 0.001), and 63.76 (68.45% alleviation, *p* < 0.001), respectively. These findings indicate that MOSY PHYCOLLREPAIR significantly mitigates UVB‐induced damage in a dose‐dependent manner, underscoring its potential as an effective UV protection agent for cosmetic applications.

### 3.9 MOSY PHYCOLLREPAIR Exhibited Melanin Inhibition in Zebrafish Embryos

**FIGURE 10:**
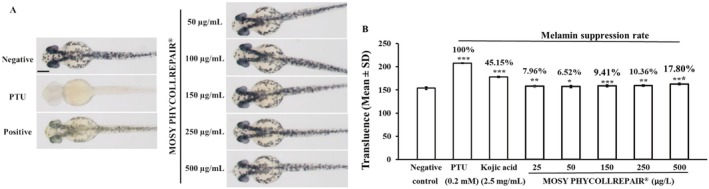
MOSY PHYCOLLREPAIR inhibited melanin synthesis in zebrafish embryos. Scale bar = 50 μm. Asterisks denote significance compared to the negative control: *p < 0.05; **p < 0.01; ***p < 0.001.

Representative images from the zebrafish embryo whitening experiment (Figure 10A) show distinct melanin signals in normal embryos (negative control). In contrast, 30 μg/mL PTU completely inhibited melanin synthesis, resulting in no visible melanin signal. The positive control, 2.5 mg/mL kojic acid, significantly reduced melanin signal compared to the negative control. However, no obvious visible melanin signal differences were observed between MOSY PHYCOLLREPAIR treatments and the negative control.

Quantitative measurements of embryo translucence (Figure 10B) revealed the following values: 153.75 for normal embryos (negative control), 207.89 (100% melanin inhibition, *p* < 0.001) for 30 μg/mL PTU‐treated embryos, and 178.19 (45.15% melanin inhibition, *p* < 0.001) for 2.5 mg/mL kojic acid‐treated embryos (positive control). For MOSY PHYCOLLREPAIR treatments, the translucence values were 158.06 (7.96% melanin inhibition, *p* = 0.009), 157.28 (6.52% melanin inhibition, *p* = 0.025), 158.84 (9.41% melanin inhibition, *p* = 0.0009), 159.36 (10.36% melanin inhibition, *p* = 0.0015), and 162.84 (16.80% melanin inhibition, *p* < 0.001) at concentrations of 25, 50, 150, 200, and 500 μg/mL, respectively. These results indicate that MOSY PHYCOLLREPAIR possesses moderate whitening activity, with the highest efficacy observed at 500 μg/mL.

## 4 Discussion

Bee honey is widely recognized for its health benefits, particularly in food and cosmetic applications. In cosmetics, honey‐based products are often used to treat problematic and damaged skin, capitalizing on their antimicrobial properties and wound‐healing capabilities [7, 8, 12, 13]. However, the general skincare efficacy of honey‐containing cosmetics has been underexplored in the literature [15]. The high glucose and fructose content (> 70%) in honey presents formulation challenges due to its low glass transition temperature, high viscosity, and hygroscopic nature, which can make products sticky and unstable if not properly formulated. This stickiness has historically limited honey's application as a general efficacy ingredient in cosmetics.

This study pioneers a novel approach to transforming honey into a more favorable cosmetic ingredient through fermentation. By combining bifidobacterium fermentation technology with low‐temperature microjet extraction, we successfully converted granulated blank bee honey into a clear, amber‐colored, slightly viscous liquid, which is highly suitable for cosmetic formulations.

Zebrafish embryo analysis confirmed that MOSY PHYCOLLREPAIR exhibits low toxicity. Further efficacy analyses revealed its multifaceted skincare benefits. Specifically, MOSY PHYCOLLREPAIR demonstrated up to 62.48% dose‐responsive antioxidant activity, up to 27.84% anti‐inflammatory activity, 295.67% ATP enhancing activity, and significant upregulation of type I collagen genes (*col1a1a*, *col1a1b*, and *col1a2*), elastin gene (*elna*), and longevity gene *sirt1*, with increases of up to 281.61%, 165.14%, 206.64%, 174.03%, and 88.24%, respectively. Additionally, it enhanced wound healing rates by up to 19.44%, provided up to 70.26% skin barrier protection against lactic acid peeling treatment, offered up to 68.45% UVB irradiation protection, and inhibited melanin production by up to 17.80%. These findings align with previous studies highlighting honey's antioxidant properties, attributed to its rich content of phenolic acids and flavonoids [3, 6, 8, 32, 33].

The bioactive compounds in honey, including phenolic acids, flavonoids, procyanidins, anthocyanins, vitamin C, vitamin E, carotenoids, enzymes (e.g., catalase, peroxidase), Maillard reaction products, and trace elements, contribute to its anti‐inflammatory, wound‐healing, and skin barrier‐enhancing properties [32, 33, 34]. These components collectively support honey's well‐documented wound repair function for problematic skin (Alangari et al., 2016; [8, 11, 12, 13]). The UVB irradiation protection activity is consistent with previous in vitro studies using UVB‐irradiated HaCaT cells [35]. The upregulation of type I collagen, elastin, and sirt1 genes likely stems from the high amino acid content in honey [12], while the rich bioactive compounds explain the upregulation of the SIRT1 gene. Collectively, these data underscore MOSY PHYCOLLREPAI's multifaceted skincare efficacy, particularly in anti‐aging, protection, and skin barrier enhancement, highlighting the success of the honey fermentation technology.

The comprehensive skincare efficacy demonstrated in this study underscores the value of further investigating MOSY PHYCOLLREPAI's composition and bioactive components. Future research should focus on both short‐ and long‐term skincare efficacy analyses of cosmetics containing MOSY PHYCOLLREPAIR to fully harness its potential in cosmetic formulations.

## 5 Conclusion

The MOSY PHYCOLLREPAIR developed through the bifidobacterium fermentation and the low‐temperature microjet extraction technologies exhibited 8 types of skin care efficacies through zebrafish embryo experiments. The MOSY PHYCOLLREPAIR is now being investigated to find the bioactive compound composition, and also being applied to cosmetics formulation. We are looking forward to evaluating its skin care efficacy on humans.

## Author Contributions

S.B. conceived and coordinated the study and proofread the paper; J.C. performed and analyzed the experiments; X.C. designed, analyzed, and wrote the paper. All authors reviewed the results and approved the final version of the manuscript.

## Ethics Statement

Zebrafish‐related operations were approved by the Department of Health, Hong Kong, SAR, China 23–90 (No. 23–86 in DH/HT&A/8/218 Pt 524.10.2023).

## Conflicts of Interest

The authors declare no conflicts of interest.

## Data Availability

Research data are not shared.
